# Disruption of Kidney–Immune System Crosstalk in Sepsis with Acute Kidney Injury: Lessons Learned from Animal Models and Their Application to Human Health

**DOI:** 10.3390/ijms23031702

**Published:** 2022-02-01

**Authors:** Kaice LaFavers

**Affiliations:** Division of Nephrology and Hypertension, Department of Medicine, Indiana University School of Medicine, Evansville, IN 47708, USA; klafaver@iupui.edu; Tel.: +1-317-374-7453

**Keywords:** sepsis, acute kidney injury, immune crosstalk, erythropoietin, vitamin D, uromodulin

## Abstract

In addition to being a leading cause of morbidity and mortality worldwide, sepsis is also the most common cause of acute kidney injury (AKI). When sepsis leads to the development of AKI, mortality increases dramatically. Since the cardinal feature of sepsis is a dysregulated host response to infection, a disruption of kidney–immune crosstalk is likely to be contributing to worsening prognosis in sepsis with acute kidney injury. Since immune-mediated injury to the kidney could disrupt its protein manufacturing capacity, an investigation of molecules mediating this crosstalk not only helps us understand the sepsis immune response, but also suggests that their supplementation could have a therapeutic effect. Erythropoietin, vitamin D and uromodulin are known to mediate kidney–immune crosstalk and their disrupted production could impact morbidity and mortality in sepsis with acute kidney injury.

## 1. Introduction

Sepsis is a complex syndrome of life-threatening organ dysfunction occurring as a result of the host response to infection. It is a leading cause of morbidity and mortality in critically ill patients [1], particularly in patients who also develop AKI, in whom mortality doubles to 50% [2]. Little is known about why the co-occurrence of these two conditions leads to such high mortality [3]. This lack of understanding has made it difficult to improve the care and positively alter the prognosis of patients with sepsis and kidney injury. Whereas electrolyte disturbances and the buildup of toxic metabolites during AKI are likely to play a crucial role in the high mortality of sepsis with AKI (SwAKI), it is also possible that a loss of protective molecule(s) from the injured kidney can contribute to the dire outcomes observed. In fact, it has been demonstrated that a prior history of AKI, even with complete recovery of filtration capability, increases the risk of developing long-term, severe sepsis, suggesting that the link between sepsis and AKI extends beyond the loss of kidney filtration function [4]. Here, we examine the relationship between the kidney and the immune system in health and disease to explore this link and identify areas of potential therapeutic intervention.

## 2. Pathophysiology of AKI in Sepsis

### 2.1. Introduction

**Figure 1:**
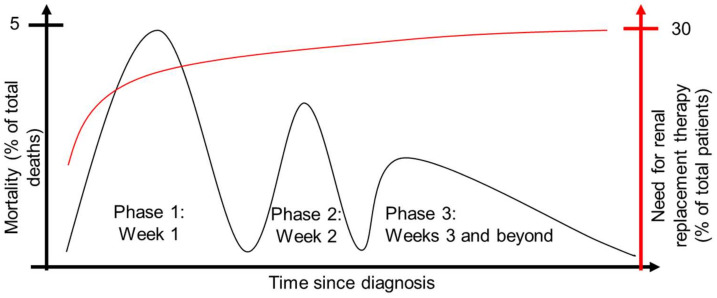
Sepsis mortality is triphasic, with three major peaks of mortality (black). The need for renal replacement therapy increases rapidly during phase 1 and only gradually thereafter (red). Based on data from [8].

The pathophysiology of sepsis is complex and very dynamic. Sepsis can be divided into an early hyperdynamic phase marked by increased cardiac output and tissue perfusion along with decreased vascular resistance. This phase is characterized by a proinflammatory state mediated by neutrophils, macrophages and monocytes (described below), followed by a hypodynamic phase marked by decreased tissue and microvascular blood flow and cardiac function, along with increasing organ injury and damage [5]. The injury to organs is likely multifactorial, caused by hemodynamic and vascular changes, direct invasion of pathogens, cytokine effects, altered metabolic programming and inadvertent consequences of a dysregulated immune response [6]. The altered immune response is a cardinal feature of sepsis and can frequently lead to a dyssynchronous and maladaptive response that worsens the injury. Immune hyperactivity will compound the injury caused by invading pathogens, whereas excessive immunosuppression will breach host defenses and typically lead to systemic collapse. For example, many immune cells maladaptively transition to apoptosis during this latter phase, with lymphocyte apoptosis occurring in the spleen and other lymphoid tissues, and apoptosis of mononuclear phagocytes throughout the body [7]. These two aspects of the sepsis immune response have led to a biphasic pattern of mortality in which patients may succumb during the early, hyperreactive stage of the illness or they may succumb during the immunosuppressive stage that follows thereafter. However, sepsis mortality remains high even beyond the time range contained within the biphasic model, with elevated mortality of sepsis patients even after apparent recovery as a result of immune dysregulation and organ damage. This has led to the revision of the biphasic model of sepsis mortality, which is now recognized to have three separate peaks (Figure 1, [8]). The need for renal replacement therapy rises rapidly from baseline to Phase 1 but increases only gradually thereafter, remaining steady at approximately 1/3 of patients having AKI severe enough to require renal replacement therapy [8].

### 2.2. Pathophysiology of Lethal Human Sepsis

In the setting of sepsis with acute kidney injury, the damage to the kidney appears to follow the sepsis themes of leukocyte infiltration and apoptosis. In the case of lethal human sepsis, apoptotic epithelial cells have been identified in the tubules of patients who succumbed to septic shock with AKI at a rate much higher than control ICU and trauma patients [9]. These patients also experienced much higher rates of capillary leukocyte infiltration and the rare presence of thrombi. In a similar study comparing postmortem kidneys from sepsis patients to kidneys from trauma/cancer patients, sepsis patients showed a dramatic increase over controls in both the percentage with focal acute tubular injury (from 15% to 78%, respectively) and the extent of that injury (1% to 10–30% of tubules injured, respectively). Electron microscopy analysis also revealed enlarged mitochondria along with an increase in the number of autophagosomes [10]. In an additional cohort of patients [11], kidney biopsies were taken from patients with sepsis-associated AKI shortly after death in the intensive care unit (ICU) and compared to nephrectomies from renal carcinoma patients. They found that sepsis AKI patients were highly heterogenous but generally showed a dramatic increase in the number of neutrophils and macrophages within the glomeruli and increased neutrophils in the tubulointerstitium. The macrophages in the AKI kidneys predominantly expressed type II markers, with a subset of macrophages expressing both type I and type II markers. In contrast, there were very few macrophages in the control kidneys. In this population, the majority of apoptotic cells were found in the tubulointerstitium, though a few AKI patients had limited evidence of apoptotic cells within the glomerulus and others did not have any apoptotic cells within the tubulointerstitium. In contrast to other studies where histological evidence of thrombi was rare, over half of the AKI patients in this study had evidence of fibrin-stained thrombi, whereas fibrin deposition was not seen in the control cancer nephrectomies. Together, these studies paint a picture of lethal human sepsis with AKI as characterized by high numbers of myeloid cells, apoptotic tubular epithelial cells and occasionally microvascular fibrin deposition with damaged mitochondria and increased autophagy occurring at the subcellular level.

### 2.3. Pathophysiology of Preclinical Animal Models of Sepsis

It has been difficult to explore the pathophysiology of non-lethal sepsis with AKI in humans as kidney biopsy is generally contraindicated in these patients. As a result, much of what we know about non-lethal sepsis with AKI comes from preclinical animal models. These models include a sterile model of endotoxemia, polymicrobial sepsis models and single pathogen models. Since the endotoxemia and polymicrobial models are the most widely used in the field of AKI research, their pathology is discussed below.

#### 2.3.1. Pathophysiology of the Lipopolysaccharide Model

The commonly used lipopolysaccharide (LPS) model leads to a consistent and reproducible inflammatory response. Sufficient doses of LPS induce renal tubular injury that is histologically characterized by necrosis of epithelial cells, loss of the brush border and tubular dilation. LPS treatment also increases cell death via apoptosis, as measured both by terminal deoxynucleotidyl transfer dUTP nick end labeling (TUNEL), as well as the upregulation of proteins associated with apoptosis, such as Apaf-1 and (cleaved) caspase-3 [12]. LPS is filtered by the glomerulus and the majority is internalized in the S1 proximal tubular segments of the kidney nephron. This uptake is dependent on the Toll-like receptor 4 (TLR4), as TLR4 knockout mice have limited uptake of fluorescently labeled endotoxin. The uptake of endotoxin in the S1 tubules then mediates increased oxidative stress and damage that is concentrated in the neighboring S2 segments and is not present in S1 segments. Using bone marrow chimeras in TLR4 and wild-type mice, the authors found that this process is dependent on renal TLR4 and is likely mediated by TNF-alpha signaling from the S1 tubules to the TNF receptor-expressing S2 tubules [13].

When a moderate dose of LPS is used (one which leads to proinflammatory cytokine release without causing shock), an antiviral response is triggered which results in a global protein synthesis shutdown [14]. Since LPS models Gram-negative sepsis, this suggests that this shutdown may occur independent of the type of pathogen initiating the infection, as this response is commonly triggered in response to viral infection. Blocking this shutdown pharmacologically decreases the kidney injury, implying that this is a maladaptive response. Given the recent nature of this work, it remains unclear whether this response extends to other preclinical models of sepsis or if it may be occurring in human sepsis as well.

Mortality is generally quite high at the doses used to study AKI in the LPS model, which has recently led to the development of a sustained endotoxemia model wherein small, repeated doses of LPS can be used to study the longer-term impact on the kidney. In this model, sustained exposure to LPS leads to macrophage infiltration and collagen deposition that continues to increase in the 3–14 days following the first injection [15]. The pathological changes induced by LPS treatment were ameliorated by rapamycin, which was also capable of reducing the ability of LPS to induce the production of inflammatory cytokines Il-1beta and MCP-1 by macrophages in vitro. This suggests that, long-term, exposure to LPS endotoxin activates mTOR signaling in macrophages, resulting in downstream kidney injury and fibrosis. Importantly, this technique could model the development of chronic kidney disease (CKD) following sepsis with AKI. 

#### 2.3.2. Pathophysiology of the Cecal Ligation and Puncture Model

The cecal ligation and puncture (CLP) model is considered by many to be the gold standard animal model of sepsis, particularly in its representation of clinical peritoneal sepsis [16]. In this model, surgery is required to open the abdomen, tie off a portion of the cecum and puncture the cecal wall, allowing fecal material to escape into the peritoneal cavity. The resulting model can be modified to modulate severity by altering the length of cecum ligated, the size/number of punctures and the timing/dosing of antibiotics to treat infection [17]. While appealing to the researcher, this makes the interpretation of CLP studies highly dependent on the exact parameters of the method. Different protocols may each more accurately represent various scenarios in clinical practice, for example, the withholding of antibiotics could model patients who do not receive prompt medical care, or the use of aged mice could provide a more accurate representation of sepsis in elderly individuals. Due to this variability, kidney pathology varies between investigators depending on the protocol used, age of mice and the timepoint after surgery. However, in mouse models with evidence of injury using late markers (i.e., serum creatinine), there may also be histological evidence of kidney injury consisting of tubular vacuolization, loss of brush borders and immune cell infiltration. Similar to injury incurred as a result of ischemia reperfusion injury, this injury can be mitigated by short episodes of ischemia and reperfusion in the limbs, known as remote ischemic reperfusion (rIPC). The rIPC can attenuate production of inflammatory cytokines and reduce apoptosis in CLP mice; this effect is dependent on the activity of HIF1α and its downstream target, mIR-21 [18]. Similar findings of apoptosis of renal cells can be seen using TUNEL staining in rat models of CLP, where they are also accompanied by histological findings of glomerular atrophy, renal capsule dilation, tubular damage and epithelial cell necrosis [19]. Despite the above findings, it is generally accepted that there is not a large amount of cell death (necrotic or programmed) in sepsis-induced AKI in comparison to other types of AKI; that damage is instead driven by inflammation, disruptions to microcirculatory flow, and metabolic perturbations in response to injury [20].

There is also evidence of autophagy in kidney tubules early on following CLP surgery, though it is already diminishing compared to sham-operated animals by 24 h [21]. More specifically, the use of autophagy to remove damaged mitochondria, known as mitophagy, is upregulated early on after sepsis in animal models and decreases over time [22]; insufficient mitophagy has been associated with worsened outcomes [23], suggesting that this process has a net benefit in sepsis. In fact, treatment of mice with rapamycin to accelerate autophagy led to increased renal function and decreased histological damage to the kidney in CLP animals [21], suggesting that increasing autophagy in sepsis could be a beneficial treatment in patients with sepsis AKI. 

**Figure 2:**
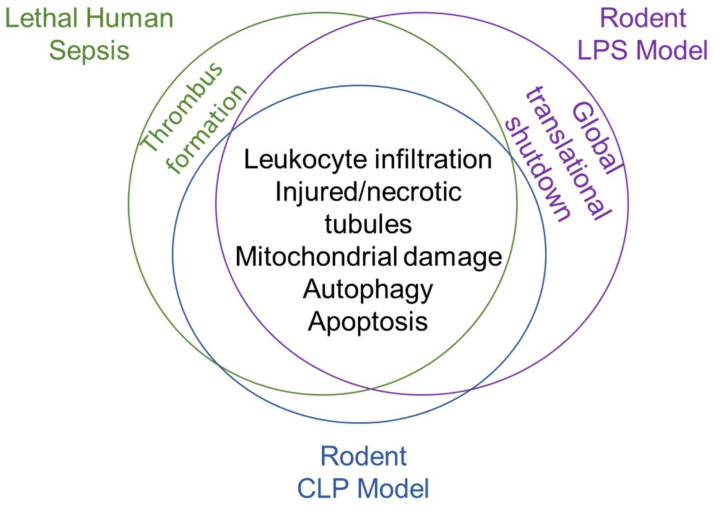
Shared pathological features of lethal human sepsis and rodent preclinical models of sepsis.

The histological renal findings in the preclinical models of sepsis support those in postmortem clinical studies (Figure 2), suggesting that they are likely to be providing an accurate glimpse into early sepsis kidney pathology. The damage downstream of oxidative stress, immune cell infiltration and overall inflammation induced within kidney sepsis is partially balanced by the “cleanup” mechanisms of the kidney, such as the use of autophagy to remove damaged organelles and cellular components. The remainder of this review will focus on the driving force in sepsis pathology, the dysregulated immune response, and how the kidney can modulate this response in adaptive and maladaptive ways in sepsis.

## 3. Immune Response in Sepsis

### 3.1. Activation of Inflammatory Signaling by Pathogen and Damage-Associated Molecular Patterns

Infections from multiple organ sites can lead to downstream sepsis once the infection is no longer confined to a single region. The spread of the infectious agent systemically will lead to the induction of an inflammatory response as immune cells recognize the invader through the interaction of pattern recognition receptors (PRRs) on their surfaces with the pathogen-associated molecular patterns (PAMPs) of the microbe (i.e., LPS, described above). Damage of host tissue can also trigger an inflammatory response through the production of damage-associated molecular patterns (DAMPs, such as heat-shock proteins, ATP, DNA) by dying cells. The presence of both PAMPs and DAMPs distinguishes sepsis from a sterile inflammatory response, which would be an immune reaction solely to DAMPs [24]. The PRRs that recognize these danger signals are primarily expressed by professional immune cells (i.e., macrophages and dendritic cells), but can be expressed by cells throughout the body [25]. PRRs are composed of multiple domains, including a ligand receptor domain and effector domain, along with (an) intermediate domain(s) that links the two [26]. The main categories of PRRs are Toll-like receptors (TLR), nucleotide-binding oligomerization domain-like receptors (NLRs), RIG-I-like receptors (RLRs), C-type lectin receptors (CLRs) and absent in melanoma-2-like receptors (ALRs). Of these, the most diverse is the TLR group, which contains thirteen members capable of recognizing a diverse array of PAMPs from bacteria, fungi, viruses and parasites. The remaining PRRs have a less broad range of pathogens which they are currently known to recognize. NLRs recognize bacterial infections, RLRs recognize viral infections, CLRs recognize fungal infections and ALRs recognize bacterial infections. Activation of PRRs leads to the activation of NF-κb, often through the action of adaptor proteins such as MyD88, which recruits proteins responsible for releasing NF-κb from inhibition by its inhibitor IκB. This allows NF-κb to translocate to the nucleus, where it is responsible for regulating the expression of genes, including pro-inflammatory cytokines (i.e., IL-1, TNFα) and DAMPs (i.e., HMGB1, CIRP, H3) [27]. In this way, the inflammatory response to an initial infectious insult is rapidly amplified. 

### 3.2. Early Production of Anti-Inflammatory Cytokines

The inflammatory response is important to contain the infectious threat [28], but it must be finely balanced to prevent too much collateral damage in cells and tissues that would otherwise remain uninjured/uninfected. As a result, anti-inflammatory cytokines, such as IL-10 and IL-1ra, are also produced early on in sepsis [29] to maintain this fine balance. In contrast to earlier dogma, the inflammatory response and anti-inflammatory response do not occur at distinct times but overlap to some extent in an effort to maintain a balanced response to the infection. If the balance swings too far in either direction, it can contribute to morbidity and mortality from either excessive inflammation and tissue/organ damage or susceptibility to further infections due to immune reprogramming [30]. In fact, one study found that levels of the both the inflammatory TNF-α as well as the anti-inflammatory cytokine IL-10 were predictive of survival in sepsis patients, with non-survivors having dramatically higher levels of both compared to survivors [29]. 

### 3.3. Long-Term Immune Derangement Following Acute Sepsis and Implications for Immunomodulatory Therapeutics

If the sepsis patient receives timely critical care, mortality can be decreased (though not eliminated) from the initial inflammatory and anti-inflammatory cytokine storms. However, complex immunological and metabolic derangements combined with sustained organ damage in these patients lead to ever-increasing mortality in patients starting 60–90 days after the initial infection [31]. These derangements may include defects in the innate immune system (i.e., persistent inflammation, a decrease in production of cytokines, immature myeloid cells, reduction in phagocytosis and antigen presentation and chronic catabolism) or in the adaptive immune system (T cell anergy, diminished T cell proliferation and cytotoxicity, increased suppression capability of Tregs and lymphocyte apoptosis). These defects can combine to result in increased rates of infection (which can be with opportunistic pathogens if adaptive immune cells have been impacted) as well as a failure of tissue to repair along with ongoing organ damage, which may result in increased risk of death. 

This suggests that a key reason why most of the clinical trials of immunomodulatory sepsis treatments have failed is that blocking either of these responses completely will simply shift the balance toward death from the compensatory response. What is instead needed are targeted therapeutics that help the patient maintain the delicate balance between the two arms of the early response while promoting recovery from the chronic immune and metabolic changes induced by acute sepsis. These therapeutics might change based on the age, symptom severity and pathogen associated with the development of sepsis. For example, the immune-suppressing corticosteroid dexamethasone significantly decreased mortality in hospitalized patients infected with SARS-CoV-2 who required respiratory support (supplemental oxygen or mechanical ventilation), but not those who did not require such support [32]. However, a meta-analysis of clinical trials for corticosteroid use in SARS-CoV-2 patients suggested this may come at the expense of increased secondary infections and delayed viral clearance [33], reinforcing the notion that treating sepsis by modulating the immune system is a delicate balancing act.

## 4. Macrophages as Drivers of Sepsis Progression

### 4.1. Macrophage Contribution to Sepsis and Septic Shock

Mononuclear phagocytes, and macrophages in particular, play a key role in the pathogenesis of sepsis [34]. Tissue macrophages are principal defenders against pathogens through phagocytosis, release of cytokines and activation of an inflammatory cascade. In an adaptive response to infections, mononuclear phagocyte activity will transition from first attacking the pathogen to “clearing the scene” and restoration of homeostasis by removing other apoptotic cells and propagating a counter-inflammatory signaling. Underlying this change in macrophage activity is a transition from a predominantly M1 phenotype to an M2 phenotype [35]. In maladaptive responses, mononuclear phagocytes can contribute to septic shock, for example through release of proinflammatory cytokines such as IL-6, IL-8, TNF-a and IL-15 [36,37], or to immune suppression through the release of anti-inflammatory cytokines such as IL-10, IL-13 and IL-4 [38,39]. Macrophages may also contribute to vascular dysfunction in septic shock due to their production of nitric oxide (NO). Macrophages produce NO through the activity of the inducible nitric oxide synthase (iNOS), which is produced only in response to inflammation by both macrophages as well as neutrophils and other cells of the body. Though NO is an excellent defense against bacterial infection, excessive amounts can lead to vasodilation, vascular hypo-reactivity and increased vascular permeability. Together, these result in the characteristic hypotension of septic shock [40]. Unfortunately, inhibition of NO production is not a viable pharmacological target. Though NOS inhibitors are capable of ameliorating hypotension in septic shock, they also exacerbate organ damage in the kidney, liver, lung, pancreas and intestines, supporting the notion that NO plays important roles in the septic response to infection that balance its negative impact on blood pressure. 

### 4.2. Loss of Macrophage Function in Sepsis Worsens Outcomes

**Figure 3:**
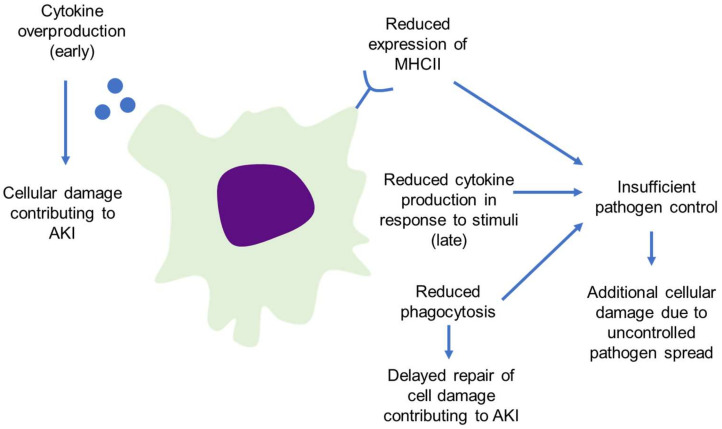
Macrophage dysfunctions linked to increased sepsis mortality.

Loss of normal macrophage function in sepsis has been known to worsen sepsis outcomes for some time. For example, the association between sepsis outcomes and MHC-II expression was first described in 1990 using flow cytometry-based approaches [41] and more recently validated using qRT-PCR [42,43]. The original studies showed that an acute decrease in MHC-II expression on monocytes following trauma is rapidly restored in patients who do not go on to develop sepsis, is more slowly restored in patients who develop sepsis and recover and is never restored in patients who succumb to sepsis [41]. This has since been expanded to other populations such that MHC-II expression, particularly the HLA-DR type, is considered to be a marker of the immune system derangement in sepsis [44] where its expression is inversely proportional to sepsis severity [43]. This suggests that the pathogen-fighting capabilities of macrophages remain incredibly important throughout the sepsis response. This is not the only function lost by mononuclear phagocytes during sepsis. Monocytes from septic individuals have a decreased capacity to secrete inflammatory cytokines (Il-1β, IL-6, TNF-α) in comparison to those isolated from healthy controls when stimulated by LPS ex vivo [45], known as endotoxin tolerance. This capacity is restored among those who survived sepsis, but not those who succumbed, suggesting that recovering macrophage function is a critical step in surviving sepsis. Phagocytic capacity has also emerged as a predictor of sepsis survival, where patients who survived infection had higher capacity for phagocytosis [46]. Phagocytic activity was measured in both monocytes and polymorphonuclear leukocytes (PMNs) isolated from septic patients using fluorescently labeled bacteria and measuring the uptake using flow cytometry. They found that phagocytosis is decreased in both monocytes and PMNs isolated on the day of admission from non-survivors when compared to survivors and controls, though the effect size is much larger in PMNs and the difference fails to reach statistical significance in the monocytes. In the surviving patients, phagocytic activity remains at similar levels on the day of discharge for both monocytes and PMNs. Together, these results suggest that maintaining optimal macrophage activity is an important driver of sepsis mortality (Figure 3) and that therapeutics which modulate macrophage activity are likely to be of benefit if they are dosed at the correct time in the course of illness.

### 4.3. Macrophage Infiltration Drives Both Damage and Repair in AKI

While improper macrophage function can drive sepsis progression due to the inability of the immune system to control the invading pathogen, macrophages can themselves drive kidney injury. The majority of macrophages that infiltrate the kidney early in the CLP model of sepsis are of the M1 phenotype, expressing high amounts of iNOS-1 and low amounts of Arg1 and FIZZ1. Preventing the accumulation of these M1 macrophages decreases kidney injury and helps maintain kidney function [47]. This is consistent with M1 macrophages role in secreting pro-inflammatory cytokines, whose action could result in cell damage/death, as described above. Conversely, M2 macrophages begin to accumulate later on in the course of sepsis and their depletion increases kidney injury and decreases kidney function, consistent with their role in repair [48]. While these studies cannot be conducted in humans kidneys due to ethical issues, circulating monocytes derived from patients in the acute versus recovery phases of sepsis show a similar monocyte plasticity in human sepsis, which was mediated by hypoxia inducible factor-1α (HIF1α) [49], suggesting that these findings may extend to septic human kidneys. 

## 5. Kidney–Macrophage Crosstalk in Sepsis

This section will explore crosstalk between the kidney and cells of the immune system, particularly macrophages. However, it is important to note that the kidney’s interaction with immune system components (i.e., immune cells and cytokines) could have impact on other organs which are beyond the scope of this review. The interested reader should consult the following review for additional information on kidney–organ crosstalk in sepsis [50].

### 5.1. Kidney Resident and Infiltrating Immune Cells

The kidneys host a wide variety of resident immune cells. Recent flow cytometry studies in uninjured human kidneys from tumor nephrectomies and kidney donor biopsies [51] found that nearly half of all Cd45^+^ cells are Cd3^+^ T cells and an additional 15–20% of Cd45^+^ cells are natural killer cells. Macrophage/monocytes and neutrophils each make up approximately 10% of the remaining, along with small (~1%) quantities of classical dendritic cells and B cells. The same authors compared this composition to young (1 month) and aged (1 year) mouse kidneys and found that mouse kidneys contain much smaller numbers of Cd3^+^ T cells (10–15% of Cd45^+^ cells) and NK cells (5–10%) along with much larger proportions of B cells (16–20%), macrophages (40–45%), scant (<1%) neutrophils and dendritic cells. It is unclear why the human kidneys contained much larger numbers of T cells as compared to mouse kidneys, though it could be due to the relative cleanliness of a mouse living in specific pathogen free conditions in comparison to a human. High proportions of T cells in human kidneys were also found, specifically in mature kidneys, within a recent scRNA-sequencing study of healthy fetal and mature kidneys [52]. By using relevant epithelial markers to determine the area from which each biopsy was likely taken (renal cortex, cortex/medulla or medulla/pelvis), they demonstrated that this T cell dominance phenomenon is especially prominent in the cortex, and the renal pelvis and myeloid cells represent a substantially larger proportion of mature immune cells within the medulla. In fetal kidneys, they found that macrophages and other myeloid cells are the dominant immune cell in development, with relative proportions of lymphocytes increasing during fetal development.

Animal studies suggest that the immune cell composition is substantially altered in the context of infection and sepsis. Recent preclinical work has shown that macrophages, along with neutrophils, make up an increasingly large proportion of Cd45^+^ immune cells within the kidney following a single bolus LPS injection, and that they are among the first responders along with endothelial and stromal cells [53]. Even as other cells are returning to baseline gene expression 48 hours after LPS administration, macrophages continue to show upregulation of genes involved in phagocytosis and cell motility along with leukotrienes, suggesting that these first responders continue to be critically important throughout the early course of sepsis progression. The expanded macrophage populations largely consisted of a group of cells lacking proliferation markers that likely represent infiltrating macrophages, suggesting that the circulation is the main source of macrophages in this setting. In the CLP model of sepsis, a recent spatial transcriptomic study revealed that infiltrating macrophages in the mouse kidney were located near proximal tubules with increased expression of the macrophage recruiting growth factor Midkine (Mdk) early on in the course of disease [54], supporting the hypothesis that the kidney recruits macrophages as front-line defenders in the context of sepsis. Transcriptomic analysis of immune cells derived from the urine of sepsis patients reveals an up-regulation in marker genes of myeloid cells concurrent with a down-regulation in T lymphocyte marker genes [55], suggesting that human kidneys also transition to a myeloid-dominant phenotype in sepsis. 

### 5.2. Kidney-Derived Molecules That Regulate Macrophage Function Could Be Key Drivers of Sepsis Progression

In addition to hosting its own resident immune cells, the kidney is the exclusive or major producer of molecules which can act directly or indirectly on the immune system. Erythropoietin, vitamin D and uromodulin were chosen as examples of such molecules due to large bodies of literature describing their immunomodulatory roles.

#### 5.2.1. Erythropoietin

The kidney is the major site of production of erythropoietin (EPO), well known for its stimulation of red blood cell production by the bone marrow in response to hypoxia, but which has recently been shown to prime macrophages for efferocytosis of apoptotic cells [56]. Erythropoietin performs its primary function by binding to its receptor, EPO receptor (EPOR), which is found on the surface of erythroid progenitor cells. This receptor is also expressed by macrophages, and the interaction of EPO with EPOR on macrophages is capable of suppressing the expression of inflammatory genes. Apoptotic cells are capable of activating EPO signaling within macrophages and promoting the removal of these dying cells by efferocytosis [56]. 

Despite the damage done to the kidney in SwAKI, plasma levels of erythropoietin are known to increase in both AKI and sepsis [57,58,59]. This increase appears to be a beneficial response, as treatment with exogenous erythropoietin or its analogues has been shown to be beneficial in multiple preclinical models of sepsis through its pleiotropic protective effects, including its ability to modulate the immune response [60,61,62,63,64,65]. While there are not currently any active clinical trials using erythropoietin for the treatment of sepsis listed on the ClinicalTrials.gov website, multiple investigators have published results from clinical trials aiming to prevent AKI in susceptible populations with mixed results [66,67,68,69,70,71], suggesting that such treatment could be beneficial in some as yet unidentified subsets of these vulnerable populations. Given the success of erythropoietin treatment in preclinical models of sepsis, it is possible that such treatment could be beneficial in some sepsis patients, though there are not any published data yet to support this. 

#### 5.2.2. Vitamin D

The kidney is also responsible for converting vitamin D3 to its active form, 1,25-dihydroxyvitamin D (1,25D) by the enzyme CYP27B1 in response to parathyroid hormone. Though other tissues are capable of producing this form, both its synthesis and its activity remain local, making the kidney an important source of systemic 1,25D. Systemic 1,25D then forms a complex with vitamin D receptors. When these are located on immune cells, they can enhance phagocytosis and chemotaxis while also suppressing proinflammatory cytokines to modulate the immune response. Monocytes are also capable of inducing their own CYP27B1 activity in response to infection, but this appears to regulate intracellular signaling that results in macrophage production of cathelicidin, a protein which can disrupt microbial membranes [72].

Vitamin D has a complex relationship with kidney injury. Low levels of vitamin D are a risk factor for AKI and are correlated with worse outcomes when AKI occurs, while toxic levels of vitamin D can cause AKI directly. AKI itself commonly leads to decreased production of vitamin D [73]. Together, these results suggest that optimal kidney health is achieved when vitamin D levels are maintained within normal limits. Supporting this, systemic levels of 1,25D are predictors of sepsis survival [72]. This is also consistent with a putative role of vitamin D as an immune modulator. As a result, many preclinical studies have been conducted using vitamin D as a treatment for various types of AKI, including sepsis-induced AKI, with promising results in mitigating both LPS-induced renal oxidative stress [74] as well as the expression of inflammatory cytokines within the kidney [75]. 

#### 5.2.3. Uromodulin

The kidney manufactures uromodulin (also known as Tamm–Horsfall protein) and releases the majority of it into the urine as a high molecular weight polymer [76]. While uromodulin has long been known to possess immunomodulatory effects in vitro [77], studies by our lab and others have found that uromodulin knockout mice have differences in the renal macrophage and neutrophil number and function, along with increased susceptibility to bladder and urinary tract infections [78,79,80,81,82,83]. The decreased susceptibility to bladder and urinary tract infections appears to be a result of physical interactions between polymeric uromodulin in the urine and bacteria. This physical interaction prevents bacteria from interacting with and forming attachments to the uroplakin receptors on the urothelial surface [80]. These apparently protective effects contrast with reports that the polymeric urinary form of uromodulin is a DAMP produced by the kidney [84] which can activate pro-inflammatory signaling pathways in myeloid cells in a TLR4-dependent manner [85]. However, a small portion of uromodulin is also secreted into the kidney interstitium and makes its way into the circulation [86]. Circulating uromodulin is not polymeric [78], suggesting that it could have very different interactions with the immune system in comparison to polymeric uromodulin. In clinical studies, both circulating and urinary uromodulin levels have been shown to be protective against kidney disease as well as cardiac and all-cause mortality, suggesting that elevated levels of uromodulin could be advantageous [87,88,89,90]. 

Interestingly, uromodulin expression in the kidney increases in the CLP model of sepsis, and initial imaging studies suggest there could be increased trafficking of uromodulin to the circulation [91]. This is in contrast to both a preclinical model of ischemia reperfusion injury AKI and a patient population with AKI in which circulating uromodulin is acutely depleted [92], suggesting that the kidney damage in CLP sepsis is not sufficient to deplete circulating uromodulin. Complete uromodulin deficiency in a knockout mouse model has also been shown to increase susceptibility to kidney injury [93], suggesting that maintaining or even increasing normal circulating uromodulin levels in acute kidney injury would ameliorate injury. In fact, treatment of uromodulin deficient mice with a non-aggregated form of uromodulin does mitigate injury in an ischemia-reperfusion model [78]. Given its ability to modulate the immune system, its renoprotective effects in AKI and its ability to mitigate renal oxidative stress [92], the increased expression and, potentially, trafficking of uromodulin to the circulation could be a protective measure by the kidney to modulate the immune system in the setting of severe infection. However, much work remains to be done to see if uromodulin is protective in the setting of sepsis and to determine its usefulness as a potential therapeutic. 

## 6. Conclusions

### 6.1. Impact of Kidney–Immune Crosstalk in Sepsis

**Figure 4:**
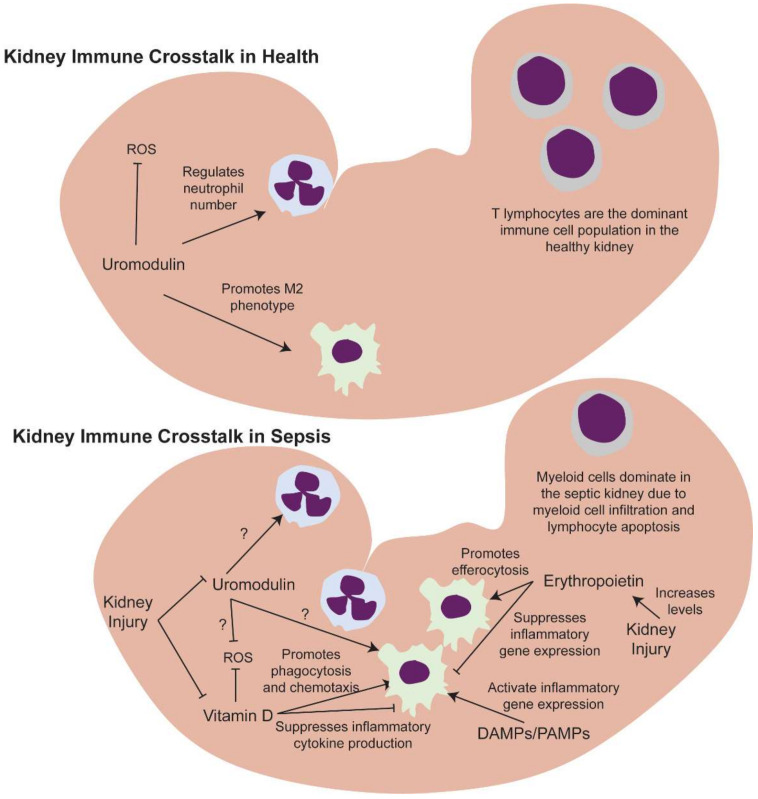
Kidney immune crosstalk in health and sepsis. Uromodulin’s immunomodulatory and anti-oxidative stress roles are well established in the healthy kidney, but it is unknown if these extend to sepsis. Vitamin D is known to promote phagocytosis and chemotaxis while suppressing inflammatory cytokine production. It can also mitigate renal oxidative stress. Erythropoeitin promotes efferocytosis and suppresses inflammatory gene expression. Damage- and pathogen-associated molecular patterns (DAMPs/PAMPs) increase in sepsis due to pathogen infiltration and cell damage. Kidney injury can modulate the levels of kidney-derived molecules. Erythropoietin increases with kidney injury while sufficient levels of kidney injury can deplete both uromodulin and vitamin D.

Together, these findings suggest a circular relationship between the kidney and the immune system in SwAKI (Figure 4). The immune response in sepsis leads to the recruitment of immune cells to the kidney, along with other organs, where the release of proinflammatory cytokines can lead to tissue damage. In a vicious cycle, DAMPs released from dead and dying cells further exacerbate the inflammatory response. Immune cells also produce anti-inflammatory cytokines that can lead to immunosuppression. If these two arms of the sepsis response are not balanced or if too much damage occurs to immune cells in the kidney and elsewhere throughout the body, this can lead to long-term immune dysregulation. Thus, it is possible for patients to experience high levels of morbidity and mortality during the initial inflammatory phase, the subsequent immune suppressed phase, and the final immune dysregulated phase.

The kidney can also produce molecules that could potentially improve sepsis outcomes, such as erythropoietin, vitamin D and uromodulin. In cases of sepsis where the damage to the cells producing these molecules is limited, it is possible that the kidney would increase or maintain their levels to modulate the immune response. However, we know that both vitamin D and uromodulin are subject to disruptions in production in AKI and these decreases could lead to improperly regulated immune responses. This suggests that supplementing their production is a potential therapeutic opportunity to improve outcomes in SwAKI. Thus, while the complex interplay between the kidney and the immune system in sepsis has negative outcomes in SwAKI, its investigation is likely to lead to beneficial advances in patient care.

### 6.2. Future Directions

Much remains to be done in the field of sepsis study, particularly for SwAKI, as morbidity and mortality associated with this condition remain unacceptably high. While clinical SwAKI remains difficult to study due to the contraindication of kidney biopsy in these patients, we can and should continue to learn valuable lessons from preclinical models and lethal human AKI. This could involve the application of novel technologies to investigate gene transcription, protein production and cellular interactions to identify new potential targets. However, there is much to be done in developing the currently identified kidney-derived molecules who could mediate crosstalk between the kidney and the immune system in sepsis. Of these, erythropoietin is the farthest along in the developmental pipeline, as it has been used as a successful treatment in preclinical sepsis models [60,61,62,63,64,65]. However, these findings have not made their way to the clinic (Table 1). Vitamin D levels are predictive of sepsis survival in patients and treatment with vitamin D is beneficial in LPS models of sepsis. Additionally, specific mechanisms of action have been identified for its protective effect in this model (Table 1, [74,75]). To further develop vitamin D’s role as a potential novel sepsis therapeutic, it needs to be characterized in other preclinical animal models of sepsis before moving on to clinical trials to bring this treatment to the clinic. Finally, uromodulin, while extensively characterized as an immunomodulatory protein made exclusively by the kidney, should be characterized in preclinical animal models of sepsis and within patient populations to determine if it may have a potential therapeutic benefit as well.

## Figures and Tables

**Table 1:** Kidney-derived molecules that could mediate kidney–immune crosstalk in sepsis.

Molecule of Interest	Lessons Learned from Animal Models	Results from Human Sepsis	Future Work Needed
Erythropoietin	Primes macrophages for efferocytosis of apoptotic cells and suppresses macrophage expression of inflammatory genes [56] Levels increase in AKI and sepsis [59] Treatment with erythropoietin is protective [60,61,62,63,64,65]	Levels increase in sepsis [57,58]	Identification of sepsis patients who would benefit from erythropoietin supplementation Clinical trials of erythropoietin supplementation in sepsis
Vitamin D	Enhances macrophage phagocytosis and chemotaxis and suppresses inflammatory cytokine production [72] Treatment with vitamin D mitigates renal oxidative stress [74] and decreases expression of inflammatory cytokines in the kidney [75] in LPS	Systemic levels are predictors of sepsis survival [72]	Vitamin D treatment in other preclinical animal models of sepsis Identification of patients who would benefit from vitamin D Clinical trials of vitamin D supplementation in sepsis
Uromodulin (Tamm–Horsfall protein)	Regulates macrophage number and phagocytic function [78] Regulates neutrophil production [79] Protective against bladder and urinary tract infection [80] Depleted in ischemic AKI [92,93], but elevated in CLP sepsis [91] Treatment with uromodulin mitigates kidney injury in ischemic AKI [50]	Levels decrease in AKI [93]	Characterization of uromodulin and uromodulin treatment in animal models of sepsis Characterization of uromodulin in sepsis patient populations Identification of sepsis patients who would benefit from uromodulin supplementation Clinical trials of uromodulin supplementation in sepsis
